# Supplementary material for: Parental Attitudes, Motivators and Barriers Toward Children’s Vaccination in Poland: A Scoping Review
Source: Vaccines (Basel). 2025 Jan 6;13(1):41. doi: 10.3390/vaccines13010041 (PMC11769564; doi:10.3390/vaccines13010041)
Supplement: Supplementary file 1 [file vaccines-13-00041-s001.zip › vaccines-3370208-supplementary.pdf]

Supplementary Materials

Parental Attitudes, Motivators and Barriers Towards Children's Vaccination in Poland: A Scoping Review

Krystyna Szalast, Grzegorz Józef Nowicki, Mariola Pietrzak, Agnieszka Mastalerz-Migas, Aleksander Biesiada, Elżbieta Grochans and Barbara Ślusarska

Table S1. PRISMA-ScR checklist [23].

Section	Item	Checklist item	Reported (Section)
**TITLE**			
Title	1	Identify the report as a scoping review.	See Title
**ABSTRACT**			
Structured summary	2	Provide a structured summary that includes (as applicable): background, objectives, eligibility criteria, sources of evidence, charting methods, results, and conclusions that relate to the review questions and objectives.	See Abstract
**INTRODUCTION**			
Rationale	3	Describe the rationale for the review in the context of what is already known. Explain why the review questions/objectives lend themselves to a scoping review approach.	See Objectives
Objectives	4	Provide an explicit statement of the questions and objectives being addressed with reference to their key elements (e.g. population or participants, concepts, and context) or other relevant key elements used to conceptualise the review questions and/or objectives.	See Objectives
**METHODS**			
Protocol and registration	5	Indicate whether a review protocol exists; state if and where it can be accessed (e.g. a web address); and if available, provide registration information, including the registration number.	See Methods
Eligibility criteria	6	Specify characteristics of the sources of evidence used as eligibility criteria (e.g. years considered, language, and publication status) and provide a rationale.	See Methods
Information sources	7	Describe all information sources in the search (e.g. databases with dates of coverage and contact with authors to identify additional sources), as well as the date the most recent search was executed.	See Methods
Search	8	Present the full electronic search strategy for at least 1 database, including any limits used, such that it could be repeated.	See Methods and Supplementary Materials
Selection of sources of evidence	9	State the process for selecting sources of evidence (i.e. screening and eligibility) included in the scoping review.	See Methods
Data charting process	10	Describe the methods of charting data from the included sources of evidence (e.g. calibrated forms or forms that have been tested by the team before their use, and whether data charting was done independently or in duplicate) and any processes for obtaining and confirming data from	See Methods

		investigators.	
Data items	11	List and define all variables for which data were sought and any assumptions and simplifications made.	See Methods
Critical appraisal of individual sources of evidence	12	If done, provide a rationale for conducting a critical appraisal of included sources of evidence; describe the methods used and how this information was used in any data synthesis (if appropriate).	Not applicable
Synthesis of results	13	Describe the methods of handling and summarising the data that were charted.	Not applicable
**RESULTS**			
Selection of sources of evidence	14	Give numbers of sources of evidence screened, assessed for eligibility, and included in the review, with reasons for exclusions at each stage, ideally using a flow diagram.	See Figure 1
Characteristics of sources of evidence	15	For each source of evidence, present characteristics for which data were charted and provide the citations.	See Characteristics of studiem and Table 1
Critical appraisal within sources of evidence	16	If done, present data on critical appraisal of included sources of evidence (see item 12).	See Results and Table 3
Results of individual sources of evidence	17	For each included source of evidence, present the relevant data that were charted that relate to the review questions and objectives.	See Results
Synthesis of results	18	Summarise and/or present the charting results as they relate to the review questions and objectives.	See Results and Table 2
**DISCUSSION**			
Summary of evidence	19	Summarise the main results (including an overview of concepts, themes, and types of evidence available), link to the review questions and objectives, and consider the relevance to key groups.	See Results
Limitations	20	Discuss the limitations of the scoping review process.	See Discussion
Conclusions	21	Provide a general interpretation of the results with respect to the review questions and objectives, as well as potential implications and/or next steps.	See Conclusion
**FUNDING**			
Funding	22	Describe sources of funding for the included sources of evidence, as well as sources of funding for the scoping review. Describe the role of the funders of the scoping review.	See Sources of Support

Table S2. Complete search string for each database searched.

Search	Keywords
#1	Vaccinations OR vaccines OR immunization
#2	Child OR Children OR Preschool OR Childhood vaccines
#3	Parents OR Mother OR Father OR Child's guardian
#4	Attitudes OR decision-making OR vaccination safety OR opinions OR beliefs Acceptance OR Agreement OR Willingness OR Refusal OR Resistance OR Confidence OR Hesitancy OR Antivaxx OR Antivaxxers OR Antivaccine OR Anti-vaccine
#5	Poland OR Polish
#5	2014 OR 2015 OR 2016 OR 2017 OR 2018 OR 2019 OR 2020 OR 2021 OR 2021OR 2022 OR 2023 OR 2024
#6	#1 AND #2 AND #3 AND #4 AND #5AND #6
